# Changing the Landscape of Remediation: The Creation and Implementation of an Institution-Wide Graduate Medical Education Performance Enhancement Program

**DOI:** 10.7759/cureus.35842

**Published:** 2023-03-06

**Authors:** Tiffany Murano, Anastasia Kunac, Neil Kothari, Machteld Hillen

**Affiliations:** 1 Emergency Medicine, Columbia University College of Physicians and Surgeons, New York, USA; 2 Surgery, Rutgers University New Jersey Medical School, Newark, USA; 3 Medicine, Rutgers University New Jersey Medical School, Newark, USA; 4 Neurology, Rutgers University New Jersey Medical School, Newark, USA

**Keywords:** education, interpersonal communication, professionalism, institution, remediation

## Abstract

Purpose

Remediation is a daunting process for both residency leadership and trainees due to several factors including limited time and resources, variable processes, and negative stigma. Our objective was to transform the remediation process by creating a transparent institution-wide program that collates tools/resources, interdepartmental faculty mentors, and positive rebranding.

Methods

Education leadership across seven specialties created a process for trainees with professionalism and interpersonal-communication skills deficiencies. Formalized departmental program-based improvement plan (PIP) and an institutional house staff performance enhancement plan (HPEP) were developed by consensus of triggers/behaviors. Utilizing published literature, a toolkit was created and implemented. Trainees were enrolled in HPEP if PIP was unsuccessful or exhibited ≥1 major trigger. Wellness evaluations were incorporated into the process to screen for external contributing factors. Surveys were sent to the program director (PD), faculty mentor, and trainee one month and six months after participation.

Results

Between 2018 and 2021, 12 trainees were enrolled. Overall feedback from PDs and the trainees was positive. The main challenge was finding mutual time for the faculty mentor and trainee to meet. Six-month surveys reported no relapses in unprofessionalism. One-year follow-up of the trainees was limited.

Conclusions

Utilizing an institution-wide standardized process of performance improvement with the removal of negative stereotyping is a unique approach to remediation. Initial feedback is promising, and future outcome data are necessary to assess the utility. The HPEP may be adopted by other academic institutions and may shift the attitudes about remediation and allow trainees to see the process as an opportunity for professional growth.

## Introduction

Remediation in medical education has been defined as “the act of facilitating a correction for trainees who started on the journey toward becoming a physician but have moved off course [1].” Although the prevalence of residents requiring remediation varies among specialties, program directors (PDs) in all specialties are tasked with guiding these trainees back on track [2-7]. 

Remediation is a challenging endeavor for both residency leadership and trainees. There is variation not only in the practices of remediation among specialties but also in the terminology used to approach struggling trainees [8,9]. Although deficiencies vary among trainees, a significant amount of time, resources, and restructuring of the program's training schedule is required to improve resident performance to the point where it meets minimum standards. In addition, the process may be associated with negative stigma or stereotyping, including the term, “remediation,” itself. Given their high level of prior academic achievement, trainees may struggle to accept that their deficiencies require more work than their peers [10]. They may express strong emotional responses, such as anger, blame, or shame, which further promotes a negative attitude toward the remediation process [4].

To our knowledge, there are few institutions that have created a uniform process for all trainees utilizing faculty and resources across multiple specialties. Although the milestones for patient care and medical knowledge vary among specialties, professionalism (Prof), interpersonal and communication skills (ICS), systems-based practice (SBP), and practice-based learning and improvement (PBLI) are shared values and commonalities that allow for pooling of resources and interdisciplinary mentorship. One other institution-wide remediation program implemented at the University of Colorado School of Medicine was established in 2006 and is focused on the remediation of a spectrum of learners ranging from medical students to faculty across all core competencies [11]. In a perspectives publication, Kalet et al. [1] recommend the creation of a system-level approach to remediation that incorporates constructive alignment, supports a continuum of practices, develops institutional communities of practice of remediation, and destigmatizes remediation. 

We sought to change the landscape of remediation with two objectives in mind: (1) Create an institution-wide standardized process for remediation of trainees utilizing institutional resources; and (2) positively rebrand the process by eliminating the term “remediation” and provide institution-wide transparency.

## Materials and methods

Creation of the Housestaff Performance Enhancement Program Subcommittee (HPEPSC)

This program was developed and implemented at an academic university-based institution. A group of education leaders across seven specialties (anesthesiology, emergency medicine, internal medicine, neurology, obstetrics/gynecology, surgery, and urology) in our institution formed a graduate medical education subcommittee (HPEPSC). The HPEPSC sought to achieve its goals by investigating current best practices for remediation and creating a program for the competencies that are shared among all specialties: Prof, ICS, SBP, and PBLI. The committee agreed to address Prof and ICS in Phase 1 of the project, with SBP and PBLI to be addressed in the forthcoming Phase 2. We decided to eliminate the term “remediation” to facilitate a more positive perception of the program. The creation of the process, toolkits, and triggers was an iterative expert consensus of the committee and content expert based on our literature search.

Creation of the toolkit and the triggers

Working off the toolkit published by Regan et al. [4], professionalism behaviors were categorized into values and conduct, accountability, responsiveness to unique characteristics/needs of patients, self-awareness and betterment, and adaptability. ICS was categorized into patient-centered communication, healthcare team communication, healthcare team leadership, and documentation in the electronic medical record (EMR).

Triggers for each of the categories were created by consensus of the HPEPSC. Triggers were defined as behaviors or actions that were non-compliant with the established standards of professionalism and ICS for our institution, common milestones, and the medical field. Triggers were identified based on prior behaviors by trainees and were classified as minor, moderate, or major based on the severity of the action/behavior. Mild triggers were actions/behaviors that were present in an isolated or infrequent evaluation and warranted a conversation between the PD and the trainee. Moderate triggers were actions/behaviors that were noted in multiple evaluations or complaints, and required intervention by the residency leadership, but did not negatively impact patient care. Major triggers were actions/behaviors that were repeatedly present in multiple sources (evaluations, complaints, or reports), negatively impacted patient care, or egregiously violated professional and ethical standards (see Appendix B [12-43] and Appendix C). 

Standardizing the process

To standardize the terms used within the institution, formal remediation plans created and implemented for trainees within their training programs were termed “program-based improvement plans (PIPs).” PIPs are initiated at the discretion of the individual program and are considered internal methods of improving resident performance. The HPEPSC asks programs to implement a PIP when trainees fail to meet appropriate milestone levels and/or exhibit ≤2 minor triggers despite counseling or a minor trigger in the presence of one moderate trigger. Residency leadership performs wellness/burnout evaluations to identify any external contributing factors or circumstances. All conversations pertaining to the PIP are documented and placed in the trainee’s file, and the GME office is notified for tracking purposes only. Participation in PIP is not disclosed to future employers for trainees who have no further incidents. Trainees are unsuccessful if there are repetitive unprofessional behaviors, there is a failure to correct current behaviors, new or additional triggers are exhibited, or goals for PIP are not achieved. Unsuccessful PIPs are documented in the trainee’s file, and they are referred to the institutional house staff performance enhancement program (HPEP) (see Appendix A).

HPEP process

HPEP is implemented when the trainee is unsuccessful in a PIP, consistently fails to meet appropriate milestone levels, is more than one level below expectation on one or more Prof or ICS milestones, exhibits more than two minor triggers repetitively despite counseling, exhibits two moderate triggers, or exhibits one or more major triggers (see Appendix A). The decision to enroll the trainee in the HPEP is made in collaboration with the trainee’s PD, Clinical Competency Committee, Department Chair, and the Designated Institutional Official (DIO). The trainee is notified in writing by the PD and the HPEPSC Chair and is required to visit the institution’s resident wellness and excellence center to screen for contributing external factors. Faculty mentors are chosen by the subcommittee chair in conjunction with the PD, and focused improvement plans are created utilizing the HPEP Prof/ICS Toolkit (Appendix B [12-43] and Appendix C). All trainees in the HPEP are assigned faculty mentors outside of their department. The plans are documented in an HPEP contract signed by the PD, DIO, faculty mentor, and the trainee. The length of the HPEP cycle is determined by the individual trainee’s needs in conjunction with all participating parties.

Summative evaluations are completed for all trainees at the completion of the HPEP cycle. Trainees are considered to have successfully completed an HPEP cycle if they meet all prescribed goals and complete all required elements of the plan. HPEP participation is not disclosed to future employers for trainees who successfully complete the program. Trainees have the option to maintain contact with his/her faculty mentor following a successful HPEP cycle, but all are encouraged to continue informal monitoring within their program to ensure that there are no further deficient behaviors. Any trainee who does not meet the expectations or goals set forth in the HPEP contract is either referred for an additional HPEP cycle, placed on probation, terminated, or does not have their contract renewed by the institution. Probation, termination, and/or non-renewal are disciplinary actions and disclosed to future employers.

The confidentiality of all trainees is maintained throughout the process. The only individuals aware that a trainee is enrolled in the HPEP are their PD, Department Chair, the HPEPSC Chair, the faculty mentor, and the DIO. PDs were informed only of the number of trainees enrolled in the program on a monthly basis during GME meetings. 

Transparency

The HPEP was placed on our GME website including a description of the program, the process, and frequently asked questions.

Evaluation

Evaluations were created via an electronic survey (Survey Monkey®) by the HPEPSC for the trainees, the PDs, and the faculty mentors. Each evaluation was composed of 8-9 questions using a 5-point Likert scale (strongly agree-strongly disagree) with two questions asking for challenges or obstacles that were faced and suggestions for potential improvements to the HPEP process. These electronic evaluations were anonymous and sent to all parties one month, six months, and 12 months after the completion of the HPEP cycle. The timing of the evaluation was chosen to enable longitudinal monitoring. All evaluations were reviewed by the HPEP members and feedback was used to make improvements. Reminders to fill out evaluations were sent to all parties on a biweekly basis.

For the analysis of this program, the Institutional Review Board approved this project.

## Results

Our institution sponsors 48 ACGME-accredited residency and fellowship programs with 663 trainees. During the academic years from 2018 to 2021, 12 trainees entered an HPEP cycle for Prof and/or ICS. All 12 trainees completed the HPEP cycle. All trainees enrolled in the HPEP completed one 3-month cycle and 10/12 of the trainees did not exhibit any additional unprofessional behaviors or had any problematic communication issues following the completion of the HPEP cycle. One trainee graduated shortly after completion and the last trainee’s contract was not renewed at the end of the academic year that coincided with the HPEP cycle.

One-month evaluations

**Figure 1:**
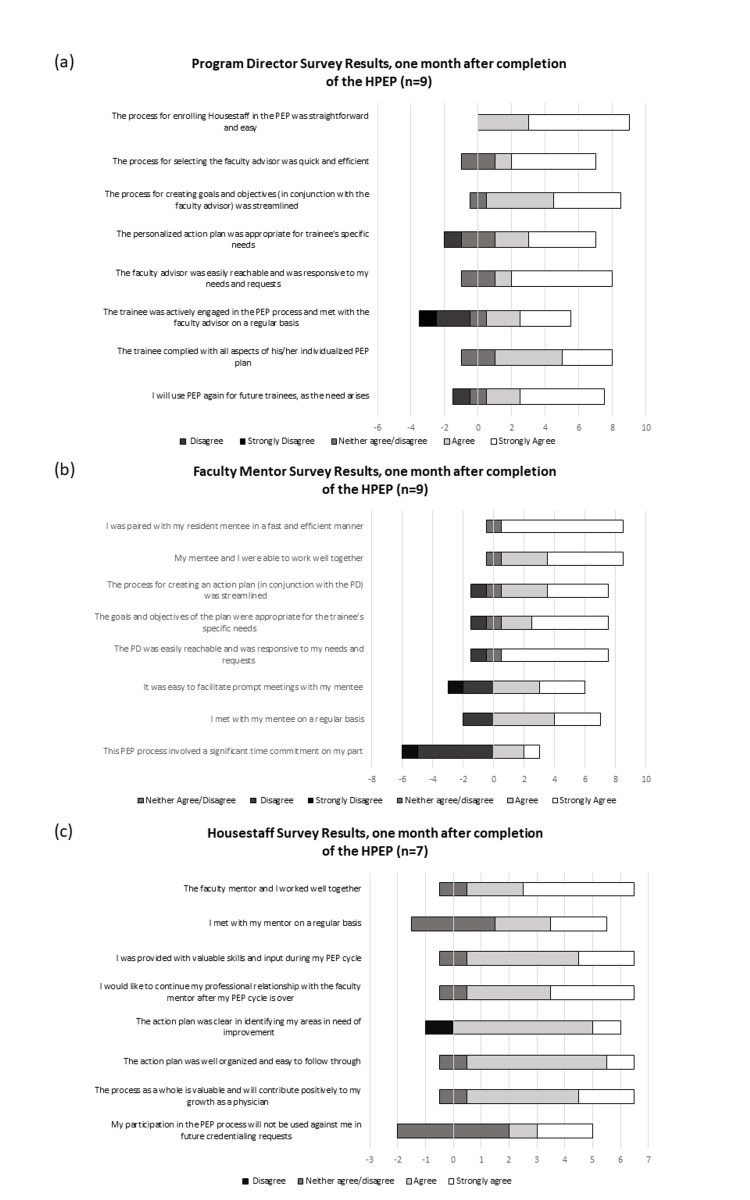
Survey Results for PDs (a), Faculty Mentors (b), and House Staff of the HPEP (c) HPEP: housestaff performance enhancement program; PEP: performance enhancement program; PD: program director.

There were two trainees who left the institution within one month of completing the HPEP. Therefore, a total of 10 evaluations were sent to the trainees with 7/10 (70%) completed. Evaluations were sent to all 12 PDs and faculty mentors with a response rate of 9/12 (75%) for both. PDs were mostly positive in their responses (Figure 1).

Of the seven trainees who responded, 85% (6/7) "strongly agreed" or "agreed" with statements pertaining to the ease and value of the process as well as the mentoring experience. When responding to the statement about whether participation in HPEP would not be used against the trainee by future credentialing requests, four of the seven trainees responded neutrally, while three agreed or strongly agreed.

Most of the faculty mentors responded "agree" or "strongly agree" to statements regarding being paired with the mentee in a fast and efficient manner and working well together with the mentee (8/9, 89%), meeting on a regular basis with the mentee, creation of a streamlined action plan and having appropriate and specific goals and objectives, and PD availability and responsiveness (7/9, 77.8%). Three mentors felt that they had difficulty facilitating meetings with the mentee and three mentors felt that the HPEP process required a significant time commitment.

Six-month evaluations

Of the 12 trainees, five did not receive the six-month evaluation because of non-renewal (1), completion of training (2), and within six months of completion of the HPEP (2). Of the 12 mentors and PDs, two did not receive six-month evaluations because their trainees were within six months of HPEP completion.

Six out of the 10 (60%) PDs responded to the six-month survey. None of the PDs referred their trainees back to the HPEP or thought that the trainees continued to meet with their mentors following the HPEP cycle. Two out of the six PDs felt the trainee continued to use the skills and tools that they learned through the PEP while three were neutral and one disagreed. Two noticed that the trainee’s behavior was and continued to be positively impacted by the HPEP process while four were neutral. Four of the six PDs felt that the trainees continued to believe that the HPEP process contributed positively to their professional development while two remained neutral. Five PDs said that they would very likely refer other trainees to the HPEP and would recommend that other PDs use the HPEP for their trainees while one PD was undecided.

One house staff responded to the six-month evaluation. Their response indicated that they did not continue to meet with the assigned faculty mentor after the HPEP. The responses to all the other statements were agree or strongly agree. Two faculty mentors responded to the six-month survey. Although only one faculty continued to meet with their mentee, both faculty agreed or strongly agreed that the trainee continued to ask for guidance after the HPEP cycle was completed and continued to use the skills and tools learned in the HPEP and their behavior continues to be positively impacted by the HPEP.

Twelve-month evaluations

Of the 12 trainees, five were unable to complete the 12-month evaluation because of non-renewal (1), completion of training (2), and within six months of completion of the HPEP (2). Of the 12 mentors and PDs, two did not receive 12-month evaluations because their trainees were within six months of HPEP completion.

Five of the 10 PDs responded to the 12-month evaluation. Two of the five PDs felt that the trainee continued to use the skills and tools that they learned, the behavior was and continued to be positively impacted, and that the HPEP process positively contributed to their professional development while three PDs were neutral in these domains. All the PDs said that they would use the HPEP if needed in the future for other trainees. All PDs reported that their trainees were not referred to the HPEP for continued professionalism or ICS issues.

Only one trainee responded to the 12-month follow-up survey. The respondent did not meet with their mentor following the HPEP but answered agree or strongly agree to other statements pertaining to altering the way they practice medicine, recommend the program to other trainees if needed, and would volunteer to participate in other areas in need of improvement.

Three of the 10 faculty mentors responded to the 12-month evaluation. One agreed that the trainee continued to meet with them and ask for guidance and feedback following the HPEP. Two positively responded that the trainees continued to use the tools and skills learned in the HPEP. Two of the mentors would recommend other faculty to serve as HPEP mentors and all three agreed or strongly agreed that they would be happy to participate as an HPEP mentor again.

## Discussion

Our program is unique in that we created a transparent institution-wide approach to assisting our trainees who did not meet minimum expectations while we positively rebranded and changed the perception of the remediation process. We wanted our trainees and faculty to see remediation as a golden opportunity, rather than a permanent blemish on their record once the trainee has graduated from residency or fellowship. Most United States’ state medical licensing boards do not require that PDs report residents who have been on remediation [44]. However, it was the impression of many PDs at our institution that remediation would negatively impact a trainee’s ability to obtain licensure.

The process, as well as the toolkit, may be helpful in institutions across all specialties to use for education in professionalism and interpersonal communication skills for trainees in their programs. Implementing a transparent process will help to create a standardized mechanism for programs to follow and perhaps allow trainees to see that the institution is committed to their learning and improvement. Jennings and Slavin [45] suggested that programs should strive for transparency whenever possible as a preventative measure to keep residents from having feelings of being treated unfairly or unjustly. In addition, this process may make institutions more attuned to how to best help their trainees and how to best utilize their resources.

Our program incorporates a wellness screen into the process by requiring trainees who are referred to the HPEP to visit the Resident Wellness and Excellence Center (RWEC). The RWEC provides confidential counseling services to the house staff at no charge and is geared toward assisting trainees with stressors that may arise inside or outside of their program. We felt that this was an important step in the process given the significant amounts of stress, burnout, and physician suicide that are affecting the medical profession on so many levels [45-49]. In a systematic review and meta-analysis by Mata et al. [48], it was demonstrated that between 20.9% and 43.2% of trainees screened positive for depression or depressive symptoms, respectively. By necessitating this meeting at the RWEC, we hope that external pressures that may be affecting a trainee’s performance begin to be appropriately addressed. We were unable to measure the success of this component due to the confidential nature of these visits. 

Overall, Phase 1 of the HPEP was well received by the trainees, mentors, and the PDs. The use of faculty mentors outside of the trainees’ specialty was implemented to allow the trainees to feel like they had a “champion” who was objective and would not be negatively influenced by interactions prior to or following the HPEP. One of the challenges that the participants of the HPEP experienced was finding time for the trainees to meet with their faculty mentors. In one case, this may have been due to the trainee’s attitude toward being in the HPEP, as they were noted by the mentor as being dismissive. However, given the demanding service schedules of both trainees and faculty, it stands to reason that the residency program should make reasonable allowances for these mentoring sessions to occur. As a few of the trainees had reading and reflective essay assignments, it is also reasonable that the training program and institution create a supportive environment to allow the participants to complete these tasks within the constructs of the functioning of the program and their scheduled work hours.

Our study has several limitations. Even though the first phase of our program has been successful in achieving its goals, the evaluation of the effectiveness of our program is limited by the small number of participants. It is also difficult to track trainees once they have graduated from the program, so longitudinal follow-up is limited. We anticipate that obtaining long-term outcomes of our program may be limited by the fact that it is difficult to follow the HPEP trainees’ performance once they have graduated from their training programs and have obtained outside employment as faculty or fellows, which would require continued contact between the program and its graduates. Further, it may be possible to assess for relapses in professionalism by querying medical licensing boards to see if the graduates had any professionalism issues that required disciplinary action. In addition, there are some unprofessional behaviors (such as sexual harassment, sexually inappropriate behavior, and substance abuse) that purposefully were not addressed in the HPEP because they are routinely referred to human resources by institution policy. With respect to the toolkit, the resources that are listed were not based on published literature and have not been proven to be helpful themselves, rather they were created for use based on the published recommendations of simulation-based case discussion. Finally, due to the COVID-19 pandemic, the HPEP process was paused as our institution was in the pandemic emergency stage for two months. The interruption in the HPEP process may have led to breakdowns in communication and loss of any previous progress that was made prior to the pandemic. In addition, six- and 12-month follow-ups may have been poor secondary to the fact that communication was hindered by the patient care demands and challenges with resources during the pandemic.

## Conclusions

The HPEP is a standardized process utilizing institution-wide resources that had positive outcomes after one year of enrollment. Rebranding the remediation process by framing it in a positive way, removing the word “remediation,” and providing transparency may eventually shift the attitudes toward remediation.

## Figures and Tables

**Figure 2:**
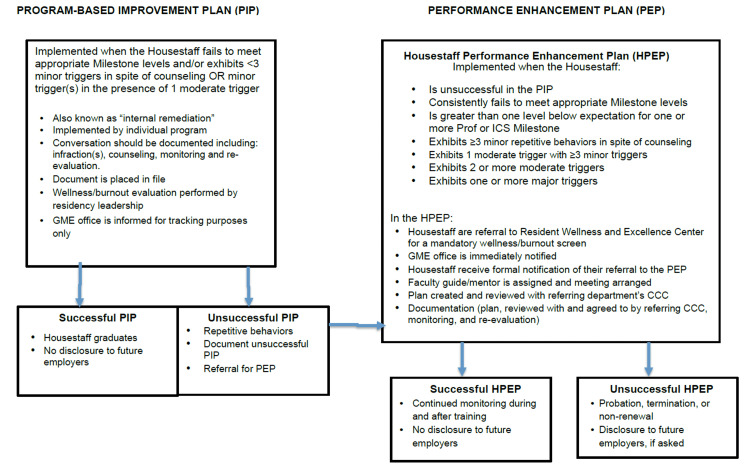
House Staff Performance Enhancement Program (HPEP) Process CCC: Clinical Competency Committee; GME: Graduate Medical Education; HPEP: Housestaff Performance Enhancement Program; PIP: Program-Based Improvement Plan.

## Appendices

Appendix A

Appendix B

**Table 1:** House Staff Performance Enhancement Program: Professionalism and Interpersonal Communication Skills Toolkit HIPAA: Health Insurance Portability and Accountability Act of 1996; PROBE: Professional/Problem-based Ethics; AMA: American Medical Association; STEPPS: Strategies and Tools to Enhance Performance and Patient Safety; EMR: Electronic Medical Record. *May require an account and/or membership for access; may get CME credit. ^Associated with tuition cost.

Milestone Category	Triggers			
	Minor	Moderate	Major	Tools
Professionalism				
Values and conduct Honesty, integrity, ethical behavior, respect and trustworthiness.	Inappropriate use of sick call. Inappropriate elevator talk. Inappropriate cell phone communication (texting or using social media).	Inappropriate use of social media. Non-HIPPA-compliant communication. Inappropriate cell phone communication in front of patient/family (texting or using social media).	HIPAA violation. Intentional deceit. Exhibits unethical behavior with research methods, statistics, data collection, documentation, or authorship. Misrepresentation of credentials (falsification of test scores, degrees, previous training).	Courses: Center for Personalized Education for Professionals PROBE^: Ethics and Boundaries program[12]. Online Courses*: AMA: Code of Medical Ethics: Creating an effective and respectful learning environment [13], AMA: Code of Medical Ethics: Political Matters, Contributions, Gifts, and Social Media: Boundaries for Physicians [14], AMA: Code of Medical Ethics: Sexual and Romantic Boundary Violations [15].
				Online resources: HIPPA Online Resources: Educational Tools for Providers [16]. Read the “Recommendations for the conduct, editing and publication of scholarly work in medical journals” [17].
				Reflective Assignments: Assign and review case scenarios (see Appendix C and Ref [18]). Read HIPAA laws and institutional compliance material and prepare a learning module.
Accountability punctuality, professional appearance and hygiene, pursuit of professional development. Complies with administrative responsibilities and has a sense of duty.	Tardiness. Poor hygiene. Inappropriate attire. Doesn’t respond to emails, pages in an appropriate time frame.	Unresponsive to emails, texts and other correspondence. Deficiencies in duty hour, patient case logs, training modules.	Untruthfulness. Leaves the premises without notifying seniors/faculty/supervisors. Patient abandonment	Courses: Center for Personalized Education for Professionals. PROBE^: Ethics and Boundaries program [12].
				Reflective Assignments: Assign and review case scenarios (see Appendix C and Ref [18]). Have trainee complete Eisenhower’s Time Matrix to assist with prioritizing tasks by urgency and importance [19]. Review Steven Covey's Time Management Matrix Explained [20,21]. Study “The 7 habits of highly successful people” [22]. Review Institutional Policy addressing attire/hygiene. Topics for trainee to review and discuss with faculty mentor: personal calendar development and email organization.
				Mentored reading program/book club: “The 7 habits of highly effective people” [22], “Peak performance” [23], “The productivity project-accomplishing more by managing your time, attention, and energy”[24], “Deep work-rules for focused success in a distracted world” [25]. Online resource: Medscape: 12 smart time management tips for doctors [26].
				Assign one or more of the following videos: Ted Talk: The Power of Vulnerability [27], YouTube: How to speak so that people want to listen [28], YouTube: The 7 habits of highly successful people [22].
Responsiveness to unique characteristics/needs of patients. Embraces cultural competency, humanism and compassion.	Doesn’t attempt to get interpreter/translator. Lack of compassion for social/economic status. Exhibited judgmental behavior in front of peers and staff.	Exhibited judgmental behavior in front of patients. Receives 1 patient complaint in regard to professionalism.	Made a decision based on personal biases which impacts patient care (will not treat someone based on biases/beliefs). Used demeaning language directly to a patient. Receives >1 patient complaint in regard to professionalism. Exhibited unprofessional behavior toward patient or staff based on gender, race, or sexual preference.	Courses: Center for Personalized Education for Professionals. PROBE^: Ethics and Boundaries program[12]. Online Course*: AMA: Code of Medical Ethics: Making decisions when professional and personal values diverge[29].
				Reflective Assignments: Assign and review case scenarios (see Appendix C and Ref [18]). Self-reflection on difficult cases highlighting micro-aggressions and bias with subsequent discussion/written essay. Simulation/Resources for LGBTQ Education [30,31].
				Have resident shadow a social worker for a defined amount of time to help gain patient perspective.
Self-awareness and betterment. Utilizes knowledge of strength and limitations, practices reflection, open to feedback. Recognizes fatigue and stress.	Resistant/defensive to feedback.	Disappeared/left patient duties unattended without affecting patient care or significant impact on patient outcome. Nodded off without impacting patient care.	Disappeared/left patient duties unattended resulting in an adverse patient outcome or significantly impacting patient care. Fell asleep affecting patient care.	Reflective assignments: Assign and review case scenarios (see Appendix C and Ref [18]). Have the trainee fill out a blank credentialing form for your hospital as if she/he were you filling it out with regard to their own performance.
Adaptability Accepts ambiguity and utilizes resources when dealing with uncertainty.	Doesn’t ask for help when needed. Doesn’t go through appropriate chain of command/channels. Doesn’t review literature, refer to text to confirm treatment, procedures, policies.	Deviation from patient care but did not impact patient care/outcome.	Adverse patient outcome because did not call for assistance.	Reflective assignments: implement cases with an emphasis on graduated responsibility and shared decision-making. Assign and review case scenarios (see Appendix C and Ref [18]).
Milestone category	Triggers			
	Minor	Minor	Minor	Tools
Interpersonal communication skills				
Patient-centered communication	Doesn't introduce himself to patient/family. Doesn’t address family concerns/questions. Poor eye contact. Doesn’t keep patient abreast of updates.	Inappropriate use of humor. Use derogatory language or terms.	Disrespectful to patient and/or family. Used inappropriate language with patients (swearing). Complaints made by patient/family.	Courses^: Center for Personalized Education for Professionals. Enhanced patient communication: Building compassion, communication and trust [32].
				Reflective assignments: implement cases with an emphasis on respectful communication. Assign and review case scenarios (see Appendix C and Ref [18]). Simulated exercises with standardized patients (+/- video review).
				Literature: Martin’s Map: a conceptual framework for teaching and learning the medical interview using a patient-centered approach [33].
Health-care team communication. Demonstrates respect; effectively transitions care and relays information; exhibits responsiveness; negotiates and resolves conflict.	Minor use of inappropriate language (swearing, insensitive jokes). A single complaint from nursing staff.	Recurrent infidelity in reporting prior events, current patient status, or care plan.	Multiple complaints from multiple sources regarding inappropriate communication. Willful failure to provide or receive appropriate transition of patient care. Willful neglect of patient care - includes failure to respond to nursing pages, consults.	Courses^: Center for Personalized Education for Professionals Improving Inter-professional communication: Working effectively in medical teams[32]. Online courses*: Enroll trainee in Team STEPPS training course [34].Online Resources*: Have trainee review key elements from Team STEPPS Patient Safety Guide [35].
				Reflective assignments: Assign and review case scenarios (see Appendix C and Ref [18]). Conflict resolution: Read, summarize and discuss one or more of the following articles/books: [36-39].
				Simulation/group activities: implement conflict resolution curriculum [40,41].
Health-care team leadership. Understands and respects all members of the team, promotes collaboration; directs teams while promoting safe patient care.	Unable to connect or form collegial relationships with team members. Makes unintentional culturally insensitive comments, racial or sexually biased comments.	Demonstrates bullying behavior to junior members of the team Insensitive to needs of other team members.	Abusive behavior to team members. Intentionally demeans other members of the health-care team.	Online courses*: Enroll trainee in Team STEPPS training course [34,35]. AMA: Code of Medical Ethics: Creating an effective and respectful learning environment [14], AMA: Code of Medical Ethics: Sexual and Romantic Boundary Violations[15].
				Mentored reading program/book club: [21,36,39].
				Reflective assignments: Assign and review case scenarios (see Appendix C and Ref [18]).
				Implement an interprofessional curriculum focused on professionalism and interpersonal communication skills [41].
Documentation in the EMR. Timely, accurate, and concise completion of information, practices within boundaries of record-sharing polices.	Inaccurate or incomplete documentation.	Failure to complete EMR, dictations in a timely fashion.	Willful falsification of health records or other documentation.	Courses^: Center for Personalized Education for Professionals Medical Record Keeping Seminar[42].
				Review literature on time management: Medscape: 12 smart time management tips for doctors [26]. Recapturing time: a practical approach to time management for physicians [43].
				Reflective assignments: Assign and review case scenarios (see Appendix C and Ref [18]).

Appendix C: Professionalism/interpersonal and communication skills case scenario

The following scenarios are meant to act as a springboard for discussion with your trainee(s). They may be played out as a role play, assigned as cases for written short answers followed by discussion or discussion alone. Cases may be modified to fit individualized situations. Trainees should be encouraged to address the following questions for each scenario:

Have you encountered this situation/scenario in the past? How have you responded in the past?

Do you see any issues with this encounter?

What are the potential issues with this encounter?

What emotional reactions to this scenario do you have (if any)?

How should you respond to your colleague?

What corrective measures should be taken?

Additional questions may be created or assigned by the faculty mentor.

Professionalism

Values and conduct; honesty, integrity, ethical behavior, respect, and trustworthiness

Case A: HIPAA

You are riding on an elevator to the 15th floor of the hospital with four other individuals whom you do not know. Two of them are wearing hospital IDs but the other two are not. A colleague from another service enters on the eighth floor. After initial greetings, he says to you, “You know, I saw Mrs. Jenkins this morning and her labs look much worse. I think she may need emergent dialysis.”

Case B-1: Texting/Social Media

You are a part of a team of physicians, nurses, and medical students rounding on a patient. Your team is in the patient’s room and the attending physician is speaking to the patient and her family. Your colleague standing next to you sends you a text containing a funny meme. She sends you additional texts that cause your phone to chirp repeatedly.

Case B-2: Texting/Social Media

You are rotating in ambulatory care. You receive a text message from a colleague with detailed health information for a patient that is admitted to another hospital in your program. The text was meant for someone else and was sent without the use of encryption.

Case C: Social Media

You have completed an overnight shift and participated in a complicated resuscitation of an interesting patient. You notice on social media that one of your colleagues has posted an image of the patient’s chest X-ray and made comments regarding the resuscitation with patient identifiers evident in the image.

Case D: Ethical Behavior Regarding Research

You have written a case report on a patient with an unusual presentation of a rare disease. You have submitted your work to the attending physician who has made significant edits to the paper and has contributed to the content to the paper. You have submitted the paper for publication and have included the attending on the paper. Your friend and colleague who saw the patient with you but did not contribute to the authorship of the paper tells you that he would like to be included in the authorship of the paper since he saw the patient with you.

Accountability; punctuality; professional appearance and hygiene; pursuit of professional development; complies with administrative responsibilities and has a sense of duty

Case E-1: Responsiveness to Administrative Duties

Your department has informed you of a hospital-mandated course for all physicians and nurses. Compliance with this course is a state regulation. Your hospital administrator has sent out numerous email reminders for the course but you have had a rigorous clinical schedule and many personal obligations that you have prioritized over this course. It is now past the deadline and you still have not completed the course.

Case E-2: Responsiveness to Administrative Duties (Flip the Script)

You are the Department Chair and have been informed that all of your clinicians, including nurses, residents, and faculty, must complete an online course on HIPAA by the end of the month. You have sent out several email reminders and yet there are several residents who have not completed the course. You have personally contacted these individuals and have informed them if this is not completed within the next 24 hours, the hospital must terminate them. Twenty-four hours later, the course is still not completed by one of these individuals.

Case F-1: Tardiness

You are rotating on a service that requires you to do 12-hour shifts. The schedule is fixed for the month and the resident who follows you is repeatedly late, sometimes up to an hour. She gives multiple excuses including traffic, child-care issues, and oversleeping.

Case F-2: Failure to Complete Assigned Duties

You are assigned for jeopardy call one day. You receive a call from your chief resident that evening stating you are being called to cover a night shift for a colleague who is reportedly ill. You complete the shift, and the next day on social media you see several pictures of this “sick” colleague at a bar for another resident’s birthday.

Case G: Patient Abandonment

You are the resident on night float. You enter the hospital and are looking for the previous team to get transition of care/hand-off. You page the day team only to find that the senior resident left the hospital 3 hours ago and has not responded to pages from the nurses or the junior residents who needed his guidance regarding a critically ill patient. When you finally get in touch with him, he tells you that he had somewhere to be and that he never received the pages.

Responsiveness to unique characteristics/needs of patients; embraces cultural competency, humanism, and compassion.

Case H-1: Cultural Incompetency

Your senior resident asks you to go see a consult in the emergency department (ED). The patient speaks Portuguese and there is no one in the ED who also speaks the language. The patient says that he can understand a little Spanish; although you can speak and understand some Spanish, you are not fluent in Spanish. The ED is loud, and the translator phone is being used by another resident. The patient is in the hallway and requires an abdominal exam and potentially may require an operation.

Case H-2: Cultural Incompetency (Flip the Script)

You are visiting Mexico on vacation and develop abdominal pain in the right lower quadrant. You suspect that you may have acute appendicitis and go to the local hospital where the staff does not speak English. The physician speaks broken English but when you ask her questions, she gives you inappropriate answers and does not appear to completely understand what you are saying.

Case I: Cultural Bias

You are caring for a 30-year-old patient who is having an acute upper gastrointestinal bleed secondary to gastric ulcers. The hemoglobin is 4.2 g/dL and the vital signs are unstable. You have approached the family to consent for blood transfusion, but they tell you that they are Jehovah Witnesses and do not want to receive blood transfusions.

Case J-1: Judgmental Behavior

You have been asked to admit a patient with a history of illicit drug use for acute chest pain. The patient has been admitted to the hospital for this same reason multiple times and always leaves against medical advice prior to cardiac testing and catheterization.

Case J-2: Judgmental Behavior (Flip the Script)

You are working in the ED and want to admit a patient with a history of illicit drug use for acute chest pain. The patient has been admitted to the hospital for this same reason multiple times and always leaves against medical advice prior to cardiac testing and catheterization. You overhear the admitting resident say to the patient, “I am not admitting you because you clearly do not want to get better. You are doing this to yourself because you are addicted to drugs.”

Case J-3: Judgmental Behavior

You are caring for a patient with severe cardiac valvular damage. The patient already had a valve replacement several years ago due to endocarditis as the result of IV drug use. The patient has not used any illegal substances for five years now and is doing well in an outpatient program. The new damage to his prosthetic valve is felt to be another episode of endocarditis, but there is no evidence of any recent drug use causing it. Your attending refuses to consider surgical management, saying “We will not put in another new valve only to be destroyed again with IV drugs.”

Self-awareness and betterment; utilizes knowledge of strength and limitations, practices reflection, open to feedback; recognizes fatigue and stress.

Case K-1: Recognizes Fatigue and Stress

You have not been sleeping well and are on call for your service. You have effectively been awake for over 24 hours and feel like you need to take a nap. Your senior resident tells you to go down to the ED to see the next two consults and then finish rounding on the service.

Case K-2: Recognizes Fatigue and Stress (Flip the Script)

You are the senior resident on a busy service. You need to delegate tasks to the junior residents and call one of your junior residents to see the consults in the ED and then instruct him to finish rounding on the service. He sounds fatigued and stressed. You notice that he has appeared this way for the past week or so but he has not communicated that to you or any other members of the team.

Case L-1: Open to Feedback

This case may be tailored to fit the procedure of choice along with complications within any given specialty.

You have successfully completed doing a procedure on a patient in the ED. It was a busy day in the ED and you feel that you could have used more help from the nursing staff and colleagues. Although the procedure was successful, there were a few areas that could have been improved.  The attending comes down and evaluates the patient and then points out multiple areas for improvement and asks you what you were thinking and why you did some things the way that you did.

Case L-2: Open to Feedback, Self-reflection, Failure to Take Responsibility

You are an intern caring for a sick patient in the ICU. Your senior has to see a new admission, so he/she asks you to watch the patient closely because the blood pressure has been very low and has required many adjustments to vasopressors. After an hour without significant change, you decide to take a break in the resident conference room with your colleagues. Your patient’s blood pressure drops acutely and he/she goes into cardiac arrest while you are gone. After the incident, your attending asks what happened and how you could have improved your handling of the patient before the code, but you only state that your senior resident was not around to help you.

Case M: Feedback and Self-reflection

Write about a time when you felt that your care of a particular patient could have been improved. Include in those thoughts how you learned that your treatment could have been better, what feedback you received or would have liked to have received from the supervising faculty, peers, patient, and other colleagues (including nurses). How did the feedback you received make you feel? What would you have done differently if you had the opportunity to do it all over again with or without the desired outcome?

Adaptability; accepts ambiguity and utilizes resources when dealing with uncertainty.

Case N: Appropriate Resource Utilization

You are on your way to see a patient having nausea and vomiting when you are called by the nurse to evaluate a patient on another team who she feels “Is not looking good.” You go to see the patient and he tells you that he is not sure what is happening, but he feels like he is going to die. His vital signs are within normal parameters but he, indeed, does not look well. The attending physician is currently in the middle of a complicated procedure with the residents on that team.

Interpersonal and communication skills

Patient-Centered Communication (PCC)

These cases may be performed as a role-play with a standardized patient(s) or in a mock oral-board fashion.

PCC-1

A 30-year-old man presents to the service requesting medication for his abdominal pain. He is insistent on getting his usual dose of narcotics and is somewhat adversarial.

PCC-2

There is a multi-vehicle accident involving a family of four in one vehicle including two children under the age of six and their parents. The children are being seen in the Pediatric ED and the husband is without complaints. The wife is a patient with head trauma and is being seen by another physician. The husband has a lot of questions about the care of his wife and his children. The trainee should attempt to address his questions and concerns and let him know that he will be updated as soon as possible. The trainees should also assess any potential needs of the husband. At some point, the nurse should interrupt and let the trainee know that patient PCC-1 is asking for more pain meds and wants to see him.

PCC-3

A 40-year-old woman presents with a complaint of chest pain. At some point her cell phone should ring and she should answer it and direct her attention away from the trainee. The nurse asks the trainee about PCC-1 and PCC-2. When the trainee tries to check on the other patients/situation PCC-3 calls him back.

Case O: Patient-Centered Communication

The nurse calls you to tell you that the patient you admitted for chest pain is upset. She told the nurse that no one told her that she was going to be admitted to the hospital, and she specifically told you that she has pets at home that need to be cared for.

Health-care team communication; demonstrates respect; effectively transitions care and relays information; exhibits responsiveness; negotiates and resolves conflict.

Case: Transition of Care

You are the consult resident on a service that requires you to take call from 9 am to 5 pm and provide care to patients in the clinic one day per week. You receive a page at 4.30 pm from a number that you recognize as being from the ED and realize that it is going to be a consult. You have made plans for dinner that evening at 6.00 pm in anticipation of being out of the hospital by 5.00 pm. Your fellow resident takes overnight call that starts at 5.00 pm.

Case: Transition of Care

You are the resident on a busy service and you have made plans to go to dinner with friends from out of town at 6.00 pm. The team has been admitting patients in the ED and is running late for afternoon rounds. At 5.30 pm you call your co-resident and they say that the team won’t be able to round for another 30 minutes. They tell you to go and meet your friends and that they will just read your patients’ charts and will “figure it out.”

Health-care team leadership; understands and respects all members of the team, promotes collaboration; directs teams while promoting safe patient care.

Case: Receiving Feedback

You see a patient in the ED with a fever and a cough. You discuss the case with your attending and tell them the diagnostic and treatment plan that you have created based on your knowledge of the disease processes and patient’s history. The attending physician disagrees with your plan and tells you that you have not considered several other disease entities in your differential diagnosis and have left out key elements of the treatment of the patient. The attending also instructs you to order additional tests. You do not agree with the attending’s assessment and diagnostic/treatment plans. How do you feel about the feedback being offered to you? What is your next course of action?

Case: Delivering Feedback

You are the senior resident working on a service and are assigned to supervise junior residents. One of the junior residents has told you that there is an admission in the ED with shortness of breath who is being admitted for an acute asthma exacerbation. You ask the junior resident to list differential diagnoses for shortness of breath and find that it is quite limited. In addition, the resident forgets several key factors in the admission order that will be necessary for the patient’s hospital stay. What are you feeling right now about the situation? How do you give this feedback to the junior resident?

Case: Knowing Your Limits/Limits of Others, Being Collegial

You are the consult resident on your service and have seen several patients in the ED who require extensive time and attention. The senior resident calls you and tells you that you also need to round on patients on the floor and follow-up on patients who were seen earlier in the day. You are feeling overwhelmed and feel like you are stretched to your limit. You communicate this to your senior resident, and they tell you that this is what it is like to be the consult resident and that you are the only resident in your class who has asked for help. Your senior resident tells you that asking for help is a sign of weakness and will impose on everyone’s time.

[The case may also be flipped and played as the senior resident]

Documentation in the EMR; timely, accurate, and concise completion of information; practices within boundaries of record-sharing polices.

Case: Timely, Accurate, and Concise Completion of Information

You are asked to complete your charts by your Department Chair. You have in excess of 50 delinquent charts which are half-completed. They are difficult to complete because you do not remember the cases completely. Your EMR has “dot-phrases” or “smart phrases” that are generic and would facilitate your completion of this task.

Case: Timely, Accurate, and Concise Completion of Information (Flip the Script)

You are consulting on a patient and see that the patient was discharged from your hospital within the past few days. During that hospital stay, the patient was evaluated by one of your partners/colleagues on your service. You go to the EMR only to discover that the discharge summary has not been completed and you are not sure what the thought process was or how the patient progressed through the hospital stay. In addition, the EMR by your colleague has not been signed and is not completed.
